# Neonatal outcomes and follow-up of children born to women with pregnancy-associated cancer: a prospective observational study

**DOI:** 10.1186/s12884-023-06182-4

**Published:** 2024-01-03

**Authors:** Michele Antonio Capozza, Alberto Romano, Stefano Mastrangelo, Giorgio Attinà, Palma Maurizi, Simonetta Costa, Giovanni Vento, Giovanni Scambia, Antonio Ruggiero

**Affiliations:** 1 Pediatric Oncology Unit, Fondazione Policlinico Universitario Agostino Gemelli IRCCS, Rome, Italy; 2 Neonatology Unit, Fondazione Policlinico Universitario Agostino Gemelli IRCCS, Rome, Italy; 3 Gynecologic Oncology Unit, Fondazione Policlinico Universitario Agostino Gemelli IRCCS, Rome, Italy; 4 Dipartimento Scienze Della Salute Della Donna, del Bambino E Di Sanità Pubblica, Università Cattolica del Sacro Cuore, Rome, Italy

**Keywords:** Cancer, Pregnancy, Antineoplastic agents, Perinatal outcomes, Congenital abnormalities, Prenatal exposure delayed effects, Clinical follow-up, Children

## Abstract

**Background:**

During the last decade, there has been a growing number of cases of children born from pregnancy-associated cancer (PAC), however there are currently insufficient data on the follow up to be observed in this category of newborns. Objective of the study was to evaluate the neonatal outcomes of infants born to mother with PAC, the potential adverse effect of chemotherapy during pregnancy and the risk of metastasis to the fetus.

**Methods:**

Maternal clinical data and neonatal outcomes of child born to mothers diagnosed with PAC were collected; infants were divided into those were and were not exposed to chemotherapy during fetal life and their outcomes were compered.

**Results:**

A total of 37 newborn infants from 36 women with PAC were analyzed. Preterm delivery occurred in 83.8% of the cases. No significant differences in neonatal outcomes were found between infants who were and were not exposed to chemotherapy during pregnancy. The median follow-up period was 12 months.

**Conclusions:**

PAC treatment during the second or third trimester does not seem to be dangerous for the fetus, however infants born from PAC must be carefully evaluated for to rule out the consequences of chemotherapy and exclude the presence of metastasis. Long-term follow-up, especially in children exposed to chemotherapy, should be encouraged to obtain relevant data on long-term toxicity.

## Introduction

 Pregnancy-associated cancer (PAC) is defined as a malignancy that occurs during pregnancy or within 12 months after delivery [1] and has an incidence of 1 in 1000 pregnancies, which increases with maternal age. Malignancy that more frequently develop during pregnancy (e.g., breast cancer, cervical cancer, ovarian cancer, melanoma, thyroid cancer, lymphoma, and leukemia) are those that typically present a peak of incidence during re-productive ages [2]. However, given the delayed childbearing observed during the last few years, tumors that typically present during the fourth to fifth decade of life (e.g., lung cancer or colorectal cancer) have occurred more often during pregnancy [3, 4].

The diagnosis of PAC may be delayed considering that symptoms related to neoplasia are frequently masked by the physiological changes occurring during this period. Moreover, caregivers are often more reluctant to perform second-level diagnostic examinations given the potential risks of damage to the fetus [5].

The risk of fetal injuries due to oncologic treatment represents a considerable challenge for clinicians. The probability of fetal harm associated with chemotherapy has closely been related to gestational age (GA), drug dose and its intrinsic pharmaco-kinetic characteristics (liposolubility, molecular weight, reduced plasma protein binding, and ionization), and the presence of drug transporters in the placenta (e.g., multi-drug resistance protein and P-glycoprotein) [6]. Providing chemotherapy to childbearing women within the 4th week of pregnancy may promote either a miscarriage or a normally developing fetus (all-or-nothing phenomenon). Between the 5th and 10th week of pregnancy, however, chemotherapy may produce teratogenic effects depending on the critical period of each organ’s development. During the second and third trimester of pregnancy, the fetus receives lesser exposure to cytotoxic drugs compared to the pregnant mother, which may explain the relatively good fetal tolerance to maternal chemotherapy during this period [7].

Childbirth should be scheduled 3 or 4 weeks after the last administration of chemotherapy to minimize the risk of myelosuppression, infections, and bleeding problems in the mother and child [8, 9]. For the same reasons, chemotherapy should generally be avoided after the 35th week of gestation due to the risk of spontaneous birth during myelosuppression [10].

Limited longitudinal data is known for newborns exposed to chemotherapy in utero, and additional research into the effects of chemotherapy on fetal and neonatal development is necessary despite recent evidence supporting the safety and effectiveness of chemotherapy for cancer during pregnancy [11]. From 12–14 weeks of gestation and up until the third trimester, the evidence and recommendations now available indicate the safety of many different chemotherapeutic drugs during pregnancy. According to the drug characteristics, the majority of chemotherapeutic drugs pass through the placenta to the fetus. Therefore, due to the increased risk of fetal deformity and stillbirth during the time of organogenesis, exposure to chemotherapy is contraindicated before 12–14 weeks of gestation.Furthermore, there are currently insufficient data on the likelihood that maternal cancer will spread to the fetus; as a result, helpful follow-up procedures for the early detection of metastases have been identified.

## Materials and methods

### Study endpoint

- The endpoints of the study were:
  To evaluate the presence of maternal tumor metastases in the child born to a mother with PAC;
  to analyze the potential toxic effects of chemotherapy during pregnancy on newborns outcomes.

### Study design, inclusion and exclusion criteria

In this observational study, data from 36 pregnant women with PAC and 37 newborns (two of whom were twins), admitted at Fondazione Policlinico Universitario Agostino Gemelli IRCCS from January 2014 to December 2019, were retrospectively analyzed.

Prior to participation, pregnant women were informed regarding the aim of the project as required by Italian Law on Privacy and Safeguarding of Sensitive Data (D. Lgs n 196, 2003), after which they provided a signed informed consent (IC) form for the use of maternal data, while both parents provided a signed IC form for the use neonatal data. The project was conducted according to the principles of the Declaration of Helsinki and the study was approved by our Institutional Review Board (DIPUSVSP-25–06-2163).

Inclusion criteria were:

- Being born to a mother with PAC;
  Availability of data related to maternal oncological history;
  Availability of data related to neonatal outcomes;
  Availability of data related to children follow-up for at least one year after the birth.

Exclusion criteria were:

- Being born to a mother without PAC;
- Absence of data related to maternal oncological history;
- Absence of data related to neonatal outcomes;
- Absence of data related to children follow-up.

### Maternal clinical data and PAC related data

Maternal clinical data were collected from computerized and paper-based records within the hospital archives. PAC cases were identified according to the International Classification of Disease codes for cancer cross-referenced with codes for pregnancy [12]. For each mother with PAC, the following clinical variables were collected:

- Demographic characteristics (age at diagnosis of PAC);
- Obstetrical data (gravidity, parity, term at diagnosis of PAC, type of delivery);
- Data related to the neoplastic disease (histology, primarily affected site, presence of metastasis at diagnosis, mode of cancer detection, symptoms, staging of disease at diagnosis according to TNM Classification, placental histological examination);
- Type and duration of the treatment (type and dose of chemotherapeutic drugs, surgery).

### Neonatal outcomes and follow up

For each newborns, the following clinical variables were collected:

- Sex;
- Data related to the birth (gestational age [GA], weight at birth, APGAR score at 1 min and at 5 min of life);
- Data related to the presence and type of malformation;
- Data related to neonatal outcomes (need for respiratory assistance at birth, presence of pneumological disease, presence of jaundice, presence of hematological abnormalities, presence of intraventricular hemorrhage, presence of cardiovascular disfunction, patent ductus arteriosus, presence of prematurity retinopathy, need for admission to intensive care unit, length of stay in hospital);
- Data related to the presence of metastasis of PAC (each newborn underwent at birth cerebral echography, chest X-rays, abdominal echography). This follow-up approach is a standard procedure at our pediatric oncology center. At present, there are no specific recommendations regarding the search for metastases in neonates.
- Newborns were categorized according to GA at birth [term (≥ 37 weeks); moderate to late preterm (< 37 weeks and > 32 weeks); very preterm (< 32 weeks and > 28 weeks); and extremely preterm infants (≤ 28 weeks)].
- Birth weight percentiles were based on Italian Neonatal anthropometrics charts [13]. We defined small for gestational age (SGA), newborns weighing less than the 10th percentile for gestational age.
- To evaluate the potential effects of chemotherapy on newborns, our population was divided into newborns who were (1) and were not (2) exposed to chemotherapy during fetal life and the related neonatal outcomes were compared.

Infants born by mothers with melanoma or hematological malignancies during pregnancy underwent chest X-rays and abdominal echography at birth and 6 months, 1 year, and 2 years thereafter to exclude possible metastases. Children born from mothers who received chemotherapy that can have toxic effects on the fetus during pregnancy underwent targeted examinations as shown in Table 1.

**Table 1:** Targeted examinations in children exposed to chemotherapy during fetal life

Neoplastic agent	Clinical evaluation	Time
**Anthracyclines**	ECG and echocardiography	At birth, 6 months, and 1 year
**Platinum compounds**	Auditory brainstem response	At 3 months
**Rituximab**	Blood dosage of immunoglobulins	Every 3 months during the first year
	Antibody response to vaccinations	At 6 months, 8 months, and 1 year

### Statistical analysis

Statistical analysis was performed using R version 4.0.2 (R Foundation for Statistical Computing, Vienna, Austria; https://www.R-project.org/). Continuous data were summarized as median and interquartile range (IR), while categorical data were summarized as numbers and percentages. Comparison between groups was performed using t-student test, while associations between primary and secondary outcomes in children and chemotherapy exposure were determined using the Fisher exact test or chi-square test as appropriate. The magnitude of the association between variables and chemotherapy exposure were expressed as odds ratios with 95% confidence intervals (95% CIs). A *P* value < 0.05 was considered statistically significant.

## Results

### Maternal clinical data and PAC related data

Thirty-six pregnant women diagnosed with PAC were evaluated in the study. The median age at diagnosis of PAC was 35.1 years (IR 26–43) with a median GA at diagnosis of 23.7 weeks (IR 6–36). In the 80% of the cases the delivery was performed by cesarean section at a median GA of 34.5 week (IR 26–40). Maternal demographic and obstetrical data are reported in Table 2.

**Table 2:** Maternal demographic characteristic and obstetrical data. (^a^)

Pregnant women	*N* = 36	Non-exposed to chemotherapy	Exposed to chemotherapy
Maternal age (years)	35.1 (26–43)	35 (26–43)	35 (27–43)
Gravidity	1.94 (1–6)	1.95 (1–6)	1,85 (1–6)
Parity	1.35 (1–3)	1,34 (1–3)	1,35 (1–3)
Gestational age at diagnosis (weeks)	23.7 (6–36)	28,1 (6–36)	19,9 (6–33)
Gestational age at delivery (weeks)	34.5 (26–40)	34,6 (25,3–40,6)	34,5 (31–37)
Cesarean section	29 (80)	17 (73,9)	13 (92,8)
Vaginal delivery	7 (20)	6 (26)	1 (7)

^a^Data are presented as median (interquartile range) or number (%)

Twenty-nine women (80%) presented with a solid tumor while 7 women (20%) presented with a hematological disease (leukemia or lymphoma). Of the 36 women, 29 (80%) cancer cases were diagnosed in the presence of patient-specific symptoms, whereas the remaining 7 cases (20%) were diagnosed during the screening programs and/or obstetrical controls. Moreover, 32 women (86.5%) were diagnosed during their pregnancy, while 4 (11.1%) were diagnosed after childbirth. Among those diagnosed during their pregnancy, 1 was diagnosed during the first trimester, 17 (47.2%) during the second trimester and 14 (38.9%) were diagnosed during third trimester. Of the 29 women with solid tumor, 18 (62.1%) presented a localized form without metastasis and without lymph node invasion, 8 (27.6%) presented without metastasis but with local lymph node invasion and 3 (10.3%) presented with metastasis. Tumor invasion of the placenta was observed in only 2 cases (poorly differentiated squamous lung carcinoma and adenosquamous carcinoma of the cervix).

The treatment approach (i.e., surgery and/or chemotherapy) was decided during pregnancy or after childbirth depending on the nature and severity of the disease. Before delivery, 4 (11.1%) patients underwent surgery, 6 (16.7%) had both surgery and chemotherapy, and 8 (22.2%) received only chemotherapy. During pregnancy, 9 women with cervical or ovarian cancer received platinum chemotherapy (carboplatin 4 or 5 AUC according to maternal weight, age, and hematological values) and taxanes [Paclitaxel 175 mg/m2, mean total dose of 612 mg/m2 with a median of 3.5 administrations (range 2–5)]. Two women with breast cancer received anthracyclines [epirubicin 35 mg/m2 weekly, mean total dose of 262 mg/m2 with a median of 9 administrations (range 3–12)]. One woman with Hodgkin’s lymphoma received three cycles of ABVD regimen (doxorubicin 25 mg/m2, bleomycin 10,000 IU/m2, vinblastine 6 mg/m2, and dacarbazine 375 mg/m2 on days 1 and 15). One woman with non-Hodgkin’s lymphoma received four cycles of R-CHOP regimen (rituximab 375 mg/m2; cyclophosphamide 750 mg/m2; doxorubicin hydrochloride 50 mg/m2; vincristine 1.4 mg/m2 (maximum 2 mg) on day 1; and oral prednisone 40 mg/m2 from day 1 to day 5). One woman with acute promyelocytic leukemia received a dose idarubicin 12 mg/m2 and one cycle of all-trans retinoic acid 45 mg/m^2^/day. Table 3 summarizes the types of cancer diagnosed in mothers and the treatments they underwent.

**Table 3:** Type of cancer and maternal treatment

Type of cancer	*N* = 36 (%)
*Solid tumors*	
Ovarian cancer	8 (22.2)
Cervical cancer	8 (22.2)
Breast cancer	5 (13.9)
Lung cancer	2 (5.5)
Melanoma	2 (5.5)
Central nervous system cancer	3 (8.3)
Submandibular gland carcinoma	1 (2.8)
*Hematological malignancies*	
Lymphoma	5 (13.9)
Acute Leukemia	2 (5.5)
**Treatment**	
None	18 (50)
Surgery	4 (11.1)
Chemotherapy	8 (22.2)
Chemotherapy and surgery	6 (16.7)

### Neonatal outcomes and follow up

Thirty-seven children born to women diagnosed with PAC were evaluated in the study, two of them were twins. The median GA at childbirth was 34.5 (IR 26–40) weeks. There were 31 (83.8%) preterm deliveries, with an early and late preterm ratio of 1/7. The median birthweight was 2341 g (IR 780–3810), with 22 (59.45%) newborns weighing < 2500 g. Congenital malformations were seen in 3 (8.1%) cases (permanent alopecia; duplicate collecting system; and left ventricular hypertrophic cardiomyopathy with notes of fibroelastosis). 5 newborns (13.5%) had and APGAR score < 7. Table 4 summarized the neonatal outcomes analyzed.

**Table 4:** Neonatal data and outcomes

**Infants**	*N* = 37
**Sex**	
Males	22 (59.5)
Females	15 (40.5)
**Gestational age**	
Preterm infants	31 (83.8)
Term infants	6 (16.2)
**Birth weight**	
Low birth weight infants (< 2500 g)	22 (59.5)
Small for gestational age infants	2 (5.4)
**Congenital malformation**	3 (8.1)
**1 min APGAR score < 7**	5 (13.5)
**Need for respiratory assistance at birth**	15 (40.5)
Intubated at the first minute	2 (5.4)
Nasal Continuous Positive Airway Pressure	12 (32.4)
**Hyaline membrane disease**	10 (27)
**Bronchopulmonary dysplasia**	1 (2.7)
**Brain ultrasound alterations**	16 (43.2)
IVH grade I and II	15 (40.5)
IVH grade III or IV	1 (2.7)
**Cardiovascular alterations**	3 (8.1)
Severe left ventricular hypertrophic cardiomyopathy	1 (2.7)
Patent ductus arteriosus	2 (5.4)
Hypotension	1 (2.7)
Need for inotropic agents	1 (2.7)
**Hematological alterations in the first week of life**	2 (5.4)
Neutropenia (absolute neutrophil count < 1500/mm3);	1 (2.7)
Thrombocytopenia (platelet count < 150,000/mm3);	1 (2.7)
**Jaundice**	13 (35.1)
Phototherapy	8 (21.6)
**Retinopathy of prematurity**	8 (21.6)
**NICU hospitalizations**	15 (40.5)
Prolonged NICU stay (> 1 month)	5 (13.5)
**Mortality**	1 (2.7%)

*NICU* Neonatal intensive care unit, *IVH* Intraventricular hemorrhage

Comparing outcomes between children who were and were not exposed to chemotherapy during fetal life, no statistically significant differences were observed (Table 5).

**Table 5:** Comparison of neonatal outcomes between newborns exposed and non-exposed to chemotherapy

Variable	Categories	N	Non-exposed to chemotherapy (%)	Exposed to chemotherapy (%)	OR	95% CI^a^	*p* value
Gestational age	Term infants	6	5 (83.3)	1 (16.7)			
	Moderate to late preterm (32 to 37 weeks)	25	14 (56)	11 (44)	3.779	(0.345; 202.168)	0.531
	Very preterm (28 to 32 weeks)	4	2 (50)	2 (50)	4.17	(0.141; 351.463)	0.5
	Extremely preterm (less than 28 weeks)	2	2 (100)	0	0	(0; 116.788)	1
Birth weight	Appropriate for gestational age (AGA)	35	22 (62.8)	13 (37.1)			
	Small for gestational age (SGA)	2	1 (50)	1 (50)	1.667	(0.02; 138.621)	1
Congenital malformation	Absent	34	21 (61.8)	13 (38.2)			
	Present	3	2 (66.7)	1 (33.3)	0.812	(0.012; 17.088)	1
1 ‘Apgar score	≥ 7	32	19 (59.4)	13 (40.6)			
	< 7	5	4 (80)	1 (20)	0.374	(0.006; 4.376)	0.630
5’ Apgar score	≥ 7	37	23 (62.2)	14 (37.8)			
	< 7	0	0	0	-	-	0.139
Need of respiratory assistance at birth	Absent	22	12 (54.5)	10 (45.4)			
	Present	15	11 (73.3)	4 (26.7)	0.446	(0.078; 2.155)	0.313
Intubated at the first minute	No	35	21 (60)	14 (40)			
	Yes	2	2 (100)	0	0	(0; 8.792)	0.516
Nasal Continuous Positive Airway Pressure (nCPAP)	No	25	15 (60)	10 (40)			
	Yes	12	8 (66.7)	4 (33.3)	0.755	(0.129; 3.834)	1
Hyaline membrane disease	Absent	27	15 (55.5)	12 (44.4)			
	Present	10	8 (80)	2 (40)	0.321	(0.028; 2.058)	0.26
Bronchopulmonary dysplasia	Absent	36	22 (61.1)	14 (38.9)			
	Present	1	1 (100)	0	0	(0; 64.008)	1
Brain ultrasound alterations	Absent	21	11 (52.4)	10 (47.6)			
	Present	16	12 (75)	4 (25)	0.376	(0.065; 1.811)	0.19
Intraventricular Hemorrhage (IVH) grade I and II	Absent	22	12 (54.5)	10 (45.5)			
	Present	15	11 (73.3)	4 (26.7)	0.446	(0.078; 2.155)	0.313
IVH grade III o IV	Absent	36	22 (61.1)	14 (38.9)			
	Present	1	1 (100)	0	0	(0; 64.008)	1
Cardiovascular alterations	Absent	34	20 (58.8)	14 (41.2)			
	Present	3	3 (100)	0	0	(0; 3.949)	0.274
Patent ductus arteriosus	Absent	35	21 (60)	14 (40)			
	Present	2	2 (100)	0	0	(0; 8.792)	0.516
Hypotension	Absent	36	22 (61.1)	14 (38.9)			
	Present	1	1 (100)	0	0	(0; 64.008)	1
Need of inotropic agents	Absent	36	22 (61.1)	14 (38.9)			
	Present	1	1 (100)	0	0	(0; 64.008)	1
Hematological alterations in the first week of life	Absent	35	21 (60)	14 (40)			
	Present	2	2 (100)	0	0	(0; 8.792)	0.516
Neutropenia (absolute neutrophil count < 1500/mm3)	Absent	36	22 (61.1)	14 (38.9)			
	Present	1	1 (100)	0	0	(0; 64.008)	1
Thrombocytopenia (platelet count < 150,000/mm3)	Absent	36	22 (61.1)	14 (38.9)			
	Present	1	1 (100)	0	0	(0; 64.008)	1
Jaundice	Absent	24	15 (62.5)	9			
	Present	13	8 (61.5)	5 (38.5)	1.040	(0.200; 5.071)	1
Phototherapy	No	29	17 (58.6)	12 (41.4)			
	Yes	8	6 (75)	2 (25)	0.481	(0.040; 3.331)	0.682
Retinopathy of prematurity	Absent	29	18 (62)	11 (37.9)			
	Present	8	5 (65.5)	3 (37.5)	0.982	(0.127; 6.288)	1
NICU/hospitalizations	No	22	12 (54.4)	10 (45.4)			
	Yes	15	11 (73.3)	4 826.7)	0.446	(0.078; 2.155)	0.313
Prolonged NICU stay (> 1 month)	No	32	19 (59.4)	13 (40.6)			
	Yes	5	4 (80)	1 (20)	0.374	(0.006; 4.376)	0.63

*N* Total number of newborns, *OR* Odds ratio, ^a^ *CI* Confidence interval, *NICU* Neonatal intensive care unit

The median follow-up period after birth was 12 months. None of the children exposed to chemotherapy during pregnancy exhibited side effects in the period of observation. Among children not exposed to chemotherapy, three children showed sideropenic anemia during their 3-month follow-up, which was treated with oral iron therapy. One child with severe left ventricular hypertrophic cardiomyopathy with fibroelastosis underwent cardiac transplantation at 6 months of age and was treated with an immunosuppressant, beta-blocker, angiotensin-converting enzyme inhibitor, and diuretic [14]. However, the observed cardiomyopathy seemed to be congenital and not related to a syndrome. One girl born at a GA of 25 weeks died at the age of 7 months due to several pulmonary, neurological, and infectious complications during NICU hospitalization.

Auditory brainstem response (ABR) was normal at 3 months of life in the nine children exposed to carboplatin, while cardiological and echocardiogram evaluation were normal at birth and 6 and 12 months thereafter in the five children exposed to anthracyclines. Immunoglobulin levels and antibody response to vaccinations were normal in the child exposed to rituximab during the third trimester of pregnancy [15]. Moreover, auxological parameters, psychomotor development, and hematological and renal hepatic functions were all normal during follow-up.

## Discussion

The current study evaluated 37 children born to mothers with PAC. Mothers were affected by solid and hematological malignancies, with an over-representation of gynecological cancers (8 ovarian and 8 cervical), as our hospital is a referral center for gynecological malignancies.

Concerning therapeutic strategy, women included herein underwent surgery and/or chemotherapy. About chemotherapy, during pregnancy, there is an increase of the third space due to the amniotic fluid and, consequently, pregnant women are less exposed to chemotherapy compared to non-pregnant women. However, the same chemotherapy regimen prescribed for women who are not pregnant [16] had been administered in accordance with current guidelines, with drug dosage adjustments depending on the patient's pregnancy-related weight gain [17].

The timing of intra-uterine exposure to chemotherapeutic drugs is crucial for fetal outcomes. Retrospective data suggest that chemotherapy should be avoided during the first trimester given the increased risk for fetal loss and/or congenital malformations. Conversely, during the second and third trimesters, chemotherapy is considered relatively safe, although obstetrical and neonatal complications may occur more frequently. Therefore, pregnancy and fetal vitality should be closely monitored [18]. All women included herein received chemotherapy from the second trimester of pregnancy, with 5 (35.7%) and 9 (64.2%) undergoing treatment in the second and third trimester, respectively; we found that our cohort had a higher incidence of congenital malformations (8.1%) than the general newborn population [19], but it is not linked to chemotherapy. In fact, 2 of the 3 cases observed were in children born to mother who had not even been exposed to chemotherapy during pregnancy. However, one case was diagnosed with a duplicate collecting system with ureterocele from the 25th week of GA after the first cycle of chemotherapy with carboplatin. The correlation between this malformation and carboplatin exposure is unclear, with one study showing that fetal exposure to carboplatin does not seem to be associated with malformations of the urinary tract [20]. Moreover, given that the kidney begins to produce urine from the 10th week of GA, while the ureter and bladder become fully formed at 21st weeks, the association between such malformations and chemotherapy may be difficult to explain [21].

In our population, a high rate of preterm births has been registered, maybe as a result of the illness itself, its treatment, and the stress that follows a cancer diagnosis. Preterm birth is mainly secondary to CS delivery due to compromised maternal health for advanced cancer [22, 23] or to initiation of treatment [24–28]. This may be a consequence of the location of the cancer (44.4% of cases had gynecological tumors). Consequently, due to the high rate of premature birth in our series, diseases related to premature birth were observed.

Notably, our records showed that only two pregnancies had placental involvement, both of which had no malignant cells passing to the fetus. This can be explained by the type of maternal neoplasm found in both patients (i.e., cervical and lung carcinoma), which more rarely metastasize to the fetus compared to melanomas (accounting for 30% of all PACs) and hematological tumors (leukemia and lymphoma) [25]. Considering the risk of fetal metastasis, the placentas of all women with suspected metastatic tumors during pregnancy should undergo thorough gross and microscopic examination. Examination of the cord blood buffy coat for the presence of tumor cells should also be implemented based on available evidence [26].

After comparing newborns exposed to chemotherapy during pregnancy with infants of women without cancer, Amant et al. [27] suggest that prenatal exposure to maternal cancer chemotherapy does not compromise the cognitive, cardiac, or general developmental abilities of children in their early childhood. The worst cognitive outcome has been linked to prematurity but not to cancer treatment. Moreover, they showed that premature babies were born more frequently to mothers with cancer regardless of antineoplastic therapy than to mothers without this diagnosis, suggesting a possible influence of maternal cancer on fetal growth apart from prematurity. Our data confirm the results while showing no differences between children who were and were not exposed to chemotherapy during fetal life. Previous studies have highlighted major concerns regarding the effects of chemotherapy on fetal growth [28–32]. Indeed, some authors have shown that the increased risk of low birth weight, small for GA, intra-uterine death, premature births, microcephaly, mental retardation, and learning difficulties may be related to chemotherapy exposure during the last two trimesters of pregnancy [29–32]. The current study found no significant differences in birth weight between infants who were and were not exposed to chemotherapy during fetal life, confirming the results presented in Abdel-Hady et al. Moreover, our results can confirm that chemotherapy administered during the second or third trimester does not affect intrauterine fetal growth and should therefore be encouraged as a treatment for pregnant women diagnosed with cancer [28]. Concerning hematological disorders, other studies [33, 34] found that some children born to mothers treated with chemotherapy presented transient myelosuppression, such us leukopenia (white blood cell count < 5000/mm3) with or without neutropenia (absolute neutrophil count < 1500/mm3), anemia, or thrombocytopenia (platelet count < 150,000/mm3). However, the disorders were not observed in our population. This was probably due to appropriate chemotherapy management (i.e., avoiding chemotherapy after the 35th week of GA) and the selection of the appropriate time of delivery (i.e., 3–4 weeks after the last chemotherapy administration or 2 weeks for weekly administered chemotherapy, such as weekly paclitaxel) to minimize the risk of myelosuppression for both mother and child [35].

Although current evidence does not indicate a standardized follow-up program, children born to mothers with PAC should receive regular multidisciplinary care to be incorporated in the standard of care of mothers with cancer during pregnancy.

In our cohort, follow-up showed an increased risk of infant mortality (1–2.7%). This finding was consistent with that presented in Lu et al., which showed increased mortality in offspring of patients with PAC, in most cases, due to prematurity [36].

Data on long-term outcomes after prenatal exposure to chemotherapy have been limited. After analyzing the outcomes of 57 children exposed to chemotherapy in utero, Hahn et al. found that most children had normal development at ages 2 to 157 months [30]. A study with a median follow-up of 22.6 months by Vandenbroucke et al. on 70 children between 17 months and 18 years of age in whom full neurologic and cardiologic examination were performed, showed that intrauterine exposure to chemotherapy was generally safe for the fetus [37]. Patients included herein who were exposed to the platinum derivative carboplatin had normal ABR, complete blood counts, serum creatinine levels, and neurologic examinations at 12-month follow-up, confirming that exposure to this drug is quite safe, despite its substantial transplacental passage due to the high free drug fraction and relatively low molecular weight [38, 39].

Regarding anthracyclines, in vitro studies have suggested the possibility of trans-placental passage, although available data on animal models have shown relatively poor delivery to the fetus [40]. Only a handful of studies have investigated the effects of chemotherapy on the fetal heart and showed that anthracyclines (idarubicin, a highly liposoluble anthracycline) during pregnancy can promote acute myocardial dysfunction. In oncologic children cardiotoxicity has been associated with its cumulative dose (> 250 mg/m^2^), the child’s gender and age, and radiotherapy or other antineoplastic agents [41–44]. Our study found that children exposed to anthracyclines did not show any cardiac function or rhythm alteration at birth and 6 and 12 months thereafter. This is in accordance with the findings of Amant et al. who did not encounter cardiac ultrasound alterations in their series after following up children who received anthracycline treatment in utero (ages 9–29 years, mean 17 years) [45].

Data from the literature regarding children born to mothers who received rituximab for the treatment of hematological malignancies have shown that its administration was associated with a selective inhibitory effect on the development of newborn B-cells. However, this condition is reversible, with B-cell levels returning to normal by 3 to 6 months of age [46]. In our study, the child exposed to rituximab during fetal life did not present any significant infections, consistent with that reported in the literature [47, 48], while subsequent immunological assessments revealed normal immuno-globulin levels and adequate vaccination response.

Some limitations of the current study include the small number of cases, which decreases our study’s statistical power, the lack of specific guidelines, and the short follow-up duration, which can be related to the retrospective nature of our study. Moreover, follow-up management of this population remains to be standardized worldwide, except for infants delivered by pregnant women with gynecological tumors [49].

The strengths of the current study include our evaluation starting at birth and the management of critical issues related to prematurity by a team of neonatologists. Moreover, the long-term follow-up had been ensured by a team of pediatric oncologists.

## Conclusions

In our population, no child born to woman with PAC experienced metastasis from the maternal tumor and the treatment of maternal cancer during second or third trimester of pregnancy does not appear to have a negative impact on the fetus. Nevertheless, pregnant women should be aware of the higher risk for prematurity and small for GA compared to the general population. As such, women with PAC should be managed at a third-level hospital with a NICU.

A personalized approach must be implemented in these children and follow-up should be guided by different factors, including the nature of the maternal tumor and its stage, antineoplastic therapy administered during pregnancy, and the clinical condition of the child. The follow-up must be based on the search for possible metastases of the maternal cancer and on the exclusion of toxic effects of chemotherapy. Nevertheless, we feel it is necessary to continue follow-up until at least the first two decades of life to evaluate neurological development, school performance, sexual maturation, and reproductive capability in this group of children.

## Data Availability

The datasets used and/or analysed during the current study available from the corresponding author on reasonable request.
